# Editorial Correspondence

**Published:** 1864-04

**Authors:** 


### EDITORIAL AND MISCELLANEOUS.

*Illinois State Medical Society.*—It gives us pleasure to direct attention to the notice of the Annual Meeting of this Society, which is contained in another part of this number of the *Journal*. We hope to see every section of the State well represented. Provision is made in the Constitution of the Society whereby *all* who attend may participate in the proceedings of the meeting. At this meeting, delegates will be selected to represent the Society in the next meeting of the American Medical Association, a notice of which will also be found in this *Journal*. Let the Annual Meeting in Chicago, in 1864, be worthy the State. We assure our brethren a cordial welcome.

*The Best Diuretic.*—Dr. A. P. Davis, of Sandford, Ind., writes us that the common Hazel-nut Shrub (*Corylus Americanus?*) growing all over the Western glades, is one of the best diuretics in use. A decoction made of the young shoots and drank freely is the most convenient mode of administration.

---

*A Misappropriation of Public Funds.*—The prosecution in the Court-Martial upon Surgeon-General Hammond has recently closed without establishing one of the charges set forth in the indictment against him. It is to be regretted that a quarter of a million of the public money should have been thus expended in a vain attempt to blast the reputation of an officer whose administration of one of the most important Government Bureaux has challenged the admiration of the medical profession of this and foreign countries.—*American Med. Times.*

---

### MEDICAL MATTERS IN VICKSBURG.

[To the Editor of the AMERICAN MEDICAL TIMES.]

SIR:—The medical affairs of the Gibralter of the Mississippi, held by the Union forces since the 4th of July last, may perhaps possess some interest for the profession at large, as well as the medical department of the army.

At the time of the first occupation of the city by General Grant, the Medical Director of the Department of the Tennessee was Surgeon Madison Mills, U. S. A., who was soon succeeded by Surgeon John Moore, U. S. V. This gentleman still continues in the office, and lately made a tour of inspection of the hospitals of Vicksburg. I freely bear testimony to the efficiency and courtesy of Dr. M., during my term of service in that department. Through the politeness of my friend and pupil, Thomas C. Coxe, Medical Cadet, I am enabled to present a few facts in relation to the hospitals there.

He says:—"Hospital No. 2 has two hundred and fifty-three patients, under charge of Surgeon Hill, 20th Ohio Vols.; the 'Marine,' under charge of Surgeon Kemble, U. S. V., has two hundred and seventeen patients. The Marine is now more generally known as 'No. 3.' The McPherson Hospital, under charge of Surgeon E. Powell, 72d Ill. Vols., has one hundred and thirty. These are all the General Hospitals now in the place.....They all draw full, or nearly so, ordinary rations, while for the lighter articles of diet for the sick, they depend almost entirely upon the 'Sanitary Commission,' whose agency here is well supplied with everything needful, which they issue liberally." Fresh fish and some few vegetables and fruits can be obtained from the market.

Vicksburg, it is well known, is situated in a district of country where fruits, such as peaches, apples, plums, cherries, etc., are easily cultivated. Melons, cabbages, tomatoes, potatoes, and other products of the garden were plentiful before the breaking out of the rebellion. The grounds around the city at present, however, are necessarily but little cultivated, and the belligerent forces destroy what is produced. The Sanitary Commission do a good work in supplying this deficiency from the rich fields of the West The onion patches of Wisconsin, the potato fields of Indiana and Illinois, contribute largely, through the Commission. Here I must express my opinion on the value of the preparation called dessicated vegetables, so common in the army (an Eastern production, I believe). They are, in my estimation, when *properly cooked*, a most excellent substitute for the fresh article.

"Surgeon J. H. Boucher, U. S. V., is the Medical Director of the Corps (17th, McPherson's); but being temporarily absent with the expedition, Asst. Surg. C. D. Davis, 17th Wis. Vols., acts in his place. Brig. Gen. McArthur is Commander of the post.

"Asst. Surgeon Ridgely, U. S. A., has turned over the Purveyor's office to Dr. Morrison."

The health of the city itself has been attended to by Surg. Churchman, U. S. V., who was appointed to the position of "Health Officer" shortly after the surrender. The chaos of unhealthiness, in which Dr. C. found the city, was soon reduced to order and cleanliness. The sick and dying negroes were removed to hospitals outside of the town; the dead mules and other animals were buried or thrown into the river; the streets, lanes, alleys, cellars, and garrets were carefully and vigorously policed, and due attention was paid to the health of the citizens in every respect. A very few of the physicians remained and practiced their professions as before. Dr. C. is energetic and efficient, and performs his duties conscientiously. He is one of the few who had left the Slave States (Virginia) before the war, from feelings of repugnance to the "peculiar institution."

Dr. Powell is well known throughout the country as a contributor to the *Chicago Medical Journal*, and as a teacher, formerly, in the "Rush Medical College," of that city. My impressions, from the intercourse I had with all these gentlemen, are pleasant and lasting; and I shall long remember the laborious and exhausting service which we performed together before, during, and after the siege. Long live our faithful comrades of Vicksburg——and let me ask in conclusion, why have not some of them been promoted to higher stations, with the Generals and other military officers? Yours, etc.,

JAMES BRYAN, *Surgeon U. S. Vols.*

141 MONTAGUE ST., BROOKLYN, Feb. 27, 1864.

---

*Indigenous Drugs.*—Now that almost every article of foreign production costs at least twice as much delivered in New York as it was sold for by the London dealer, the question naturally arises—Is there no possibility of our superseding articles necessarily imported from abroad by American productions thus reducing the now enormous cost of drugs to the consumer, and restoring the greatly reduced profits of the dealer? Against the attempt to bring in an indigenous plant for use in medicine, the traditional reputation of the old and long tried *remedies* is constantly arrayed. The writings of physicians of half a century ago still furnish the staple of much of the teaching in our schools, and our text-books are occupied with elaborate descriptions of remedies, some of which are now rarely attainable, while their reputation is perhaps chiefly due to their having been "known to the ancients." The absurd adherence of our Pharmacopœia to scammony as an ingredient of our leading popular cathartic pills is an illustration of this conservatism, the practical operation of which is, that some of the officinal formulas are ignored by nine of every ten of the manufacturers who furnish our preparations; and the prices which pharmaceutists can afford to pay with a view to the popular demand being far below the possibilities of the case, we can no more blame the deficiency upon the manufacturer or upon the pharmaceutist than upon the physician or consumers themselves.

It is the misfortune of almost every good thing to be damaged by our zealous advocates, and it happens that, owing to the existence of the sect of the so-called Eclectic physicians, who seem to have founded their system of practice upon the narrow idea that a medicine must be of vegetable origin, and even that it must have grown on American soil, to merit their approval, the true importance of many of our indigenous remedies has gained ground but slowly. The empirical processes of the Eclectic manufacturers, by which the attempt is made to reduce all the indigenous remedies to a uniform form of preparation, unwarranted by a scientific appreciation of their varied composition, has also stood in the way of their reputation. Many of the so-called concentrated remedies are far from representing the drugs from which they were obtained; and any estimate of the therapeutic value of those drugs founded upon experiments with the Eclectic resinoid principles obtained from them, is liable to their real uses and adaptations.

We believe that with scientific pharmaceutists mainly rests the work of bringing the numerous American drugs which are readily obtainable, and many of foreign origin which are capable of easy propagation on our own soil, to a fair trial upon their merits; and we would suggest that every effort in this direction is not only a good business move, but tends directly to promote the prosperity and independence of our own country, and to diffuse its rich and useful productions throughout the world.—*Am. Drug. Circular.*

---

*Pulmonary Congestion in Children simulating the Early Stage of Phthisis.*—In a lecture delivered at the Hôpital des Enfants Malades, M. Bouchut summed up his remaks in the following conclusions:—

There are cases of chronic pulmonary congestion which perfectly resemble, in their physical signs, tubercle of the lungs in its first or crude stage. These congestions are asthenic, and are readily cured by the use of sulphureous waters; while true tuberculosis is much less amenable to this treatment.

Chronic pulmonary congestion is observed in children as well as in adults; it is the result of acute congestion, of bronchitis, of pneumonia, either simple or attendant on measles, of rheumatic or herpetic bronchitis, or of pulmonary apoplexy, which has not been entirely recovered from.

A kind of chronic pulmonary apoplexy, characterized by infiltration, destroying the pliability of the lung-tissue, and increasing its density, constitutes the anatomical condition of chronic pulmonary congestion.

Chronic pulmonary congestion may exist alone, and may remain so without the development of tubercle; on the other hand, it is tolerably often only the first phase of phthisis. In the same way as there are chronic hyperæmic states of the glands in children, which may or may not be followed by tubercle, so pulmonory congestion may be found to constitute the entire disease. Chronic pulmonary congestion must, however, always be looked on with suspicion, because it may be the origin of true phthisis.

Whatever be the nature of the induration of the lung—whether it be from congestion, exudation, apoplexy or tubercle—its effect will be to partially arrest the blood-changes, by impeding the access of air to the pulmonary vesicles, and will produce the same physical signs.

Chronic pulmonary congestion, in scrofulous patients, necessarily leads to phthisis; in plethoric, rheumatic, and herpetic individuals, it remains in the congested or indurated state until resolution takes place.

Nothing has so great a resemblance as chronic pulmonary congestion to the first stage of phthisis; for the physical signs are alike, and the general symptoms are almost the same. The physical signs of chronic pulmonary congestion are, relative dulness of the chest; weakening of the vesicular murmur; prolonged expiratory murmur; some mucous ronchi; and increased vocal resonance—signs generally held to be characteristic of crude tubercle in the lung. The general symptoms are cough, with or without expectoration; emaciation; and sometimes *malaise*, weakness, or a febrile state.

Chronic pulmonary congestion lasts from a few months to several years; but recovery generally takes place, unless the affection become complicated with tubercle. Pulmonary tubercle is very rarely recovered from: most of the alleged cases of recovery have in reality been c ses of pulmonary congestion. The disease is more readily recovered from in rheumatic and herpetic than in scrofulous subjects.

The treatment should consist of cod-liver oil in the winter, and of quinine-wine and arseniate of soda in the summer; and the patient should be sent to the sea-side or to the country.—*Brit. Med. Jour.*, Sept. 12, 1863, from *Gazette des Hôpitaux*, 21 July, 1863.

---

*Dietetic Treatment of Albuminuria.*—The researches of M. Hamon, contained in a note on albuminogenesis communicated to the Académie de Médicine on the 29th April, 1862, appear to throw considerable light upon the question whether any kinds of aliment increase or diminish albuminuria He found, for instance, that soft-boiled eggs were very easy of digestion, and exercised a very slight albuminogenic influence, and it has therefore been suggested that in albuminuri an albuminous diet might be ordered, composed principally of softboiled eggs and aluminous water. Vegetable diet has also been sometimes employed in albuminous anasarca, sometimes with partial, sometimes with perfect success. But all kinds of diet that have been adopted in albuminuria are inferior to that of milk, as recommended by M. Guignier. The latter physician adopts the treatment of albuminuria devised by M. Serres, and which is founded on the combination of three plans—namely, the diminution of drink, the milk regimen, and the use of raw onions. The plan consists in choosing the milk with great care as to its freshness and quality, in substituting one milk for another when the first disagrees with the patient, allowing the patients to determine the quantity they drink, uniting lime or magnesia with the milk when it causes acidity and abandoning the treatment at the end of twenty days, if it has not produced a decided good effect. M. Guignier believes that the diuretic property of the raw onion has a beneficial effect upon the disease, and that these vegetables ought to form part of the milk regimen. The editor of the *Bullétin Génèral de Thérapeutique* thinks that albuminuria may be cured by dietetic means, if they are adopted early, when the effusions are recent, and before the albuminuria has lasted long enough to have produced in the renal tissues the organic changes which place the case beyond the hope of cure.—*Brit. and For. Med-Chir. Rev.*, July, 1863, from *Bul. Gén. de Théra.*, March, 1863.

